# Agenda of Early Life Experience and Its Association with Sensitivity to Human Presence and Familiarity in Wild-Born Orphaned Captive Cheetahs

**DOI:** 10.3390/ani14223223

**Published:** 2024-11-10

**Authors:** Alessandro Gallo, Anne Schmidt-Küntzel, Lea Petersen, M. Justin Moya, Laurie Marker, Alban Lemasson, Martine Hausberger

**Affiliations:** 1 Université de Caen-Normandie Laboratoire d’Ethologie Animale et Humaine, EthoS, UMR 6552 CNRS, Université de Rennes, 35000 Rennes, France; alessandro.gallo@univ-rennes.fr; 2 CNRS, Integrative Center for Neuroscience and Cognition, INCC, UMR 8002, Université de Paris Cité, 75006 Paris, France; mhausberger.pro@gmail.com; 3 Centre de Recherche Et d’Études Pour L’Animal Sauvage (CREAS), 44710 Port Saint Père, France; 4 Cheetah Conservation Fund, Otjiwarongo P.O. Box 1755, Namibia; genetics@cheetah.org (A.S.-K.); ccfkeeper@cheetah.org (L.P.); justinm@cheetah.org (M.J.M.); director@cheetah.org (L.M.); 5 Institut Universitaire de France, 75005 Paris, France; 6 Department of Zoology and Entomology, Rhodes University, Makhanda 6140, South Africa

**Keywords:** *Acinonyx jubatus*, hand rearing, human–animal interactions, maternal loss, species-specific representation

## Abstract

It has been argued that domestication explains the ability of domestic animals to use human cues, but similar abilities exist in wild animals repeatedly exposed to humans. Little is known on the importance of the developmental stage of this exposure for developing such abilities. Orphancy and subsequent hand-rearing constitute a quasi-experimental situation for investigating this question. Cheetahs (*Acinonyx jubatus*) are interesting as they present a two-step development, spending their first two months of life in a den with occasional visits from the mother, and then accompanying their mother outdoors. Mother loss may occur at any of these stages, and the orphaned cheetahs are then taken under human care. We tested the importance of the stage of development on their long-term perception of humans by comparing the reactions of adult wild-born cheetahs, orphaned at “den-age” (Early-Orphaned, 0–2 months) or at a later stage (Late-Orphaned, 2–6 months), to motionless humans (familiar vs. unfamiliar). The results showed that Early-Orphaned, but not Late-Orphaned cheetahs, produced affiliative calls (purrs) towards humans and discriminated familiar and unfamiliar humans. Taken together, these results suggest that the timing in which an emotional bond is created with the primary caregiver (humans in the case of Early-Orphaned cheetahs and the mother in the case of Late-Orphaned cheetahs) is crucial in determining the reaction towards the species of the early caregiver throughout life.

## Simple Summary

This study investigates the importance of the timing of early experience with conspecific and human models on the long-term perception of humans. We compared the reactions of wild-born captive adult cheetahs to the presence of motionless familiar and unfamiliar humans according to their developmental history, i.e., orphancy and subsequent hand-rearing either as a young cub (at a stage when it would be hidden in a den, Early-Orphaned) or as a somewhat older juvenile (at a stage where it was accompanying its mother outdoors, Late-Orphaned). The results show clear differences, with Early-Orphaned cubs showing more affiliative behaviours, especially towards familiar humans, contrarily to Late-Orphaned cheetahs which showed none of these characteristics despite being attentive. As a whole, this suggests that the timing at which the deprivation from the primary conspecific caregiver and human hand-rearing happen can shape the future perception of humans. This also suggests that the timing in which the primary caregiver is replaced can also affect the social cognition of non-domesticated animals, thus having implications also for conservation and care in human settings.

## 1. Introduction

It has been argued that domestication (i.e., result of human control of reproduction over generations) explains the ability of domestic animals to use human cues [1], but similar abilities exist in wild animals repeatedly exposed to humans. However, the importance of the timing of these interactions during development is poorly known. Early experiences play a major role in the development of social cognition in vertebrates, including social integration and the formation of proper species-specific representations [2,3,4]. This is particularly noticeable in young animals that experience maternal loss, which manifests itself through long-term difficulty in establishing social bonds with conspecifics and a potential increase in aggressiveness [5,6,7,8,9,10]. Wild-born animals that are orphaned and rescued by conservation/rehabilitation centres need to be subsequently hand-reared by humans. These young animals thus experience both parental deprivation and interspecific fostering, two aspects that may considerably impact the development of their social and cognitive skills (e.g., decreased social motivation and communicative skills) [11,12,13,14].

Mammalian developmental psychobiology is characterized by critical phases in which interactions with the primary caregiver have a crucial role [15,16,17]. Indeed, the parental bond represents the first social feedback for the offspring and contributes to the development of proper behavioural and social competences [18,19,20]. As such, adult conspecifics have the role of “models and regulators” for immature individuals [21,22] and influence their neurogenesis and cognitive development [23,24]. Conversely, parental deprivation appears to carry negative effects on offspring development. Early-deprived individuals are characterized by altered emotional profiles, such as increased anxiety levels and impulsiveness [15,22,25,26], as well as disrupted social competencies when interacting with conspecifics (e.g., increased spatial distances and aggressive interactions, and decreased allogrooming) [5,7,8,9,21]. As a whole, interactions with adult conspecifics through selective attention act as a driving force in building appropriate species-specific and social representations in immature individuals, with visible long-term effects in adulthood [27,28,29].

In the absence of parental care, humans rearing orphaned individuals act as an interspecific foster parent. Repeated interactions with humans constitute the foundation of the development of human–animal relationships [30]. When interacting with humans, animals may learn the individual characteristics of their caretakers and be able to discriminate between familiar and unfamiliar humans on the basis of their individual cues (i.e., visual, acoustic, or olfactory) [31,32,33,34,35,36,37,38,39,40,41,42] and to associate an emotional valence to each interaction with humans [43,44]. Specifically, they may respond to a human presence with proximity seeking (positive emotional valence), avoidance (negative emotional valence), or no reaction at all (neutral emotional valence), depending on memories of past interactions (e.g., [45]). Generalization of the emotional valence to unfamiliar humans has also been demonstrated in different domestic and captive species [41,46,47,48,49,50]. Furthermore, human rearing can influence the species-specific representation, with humans becoming preferential partners. This can, for instance, be seen in the classic case of Lorenz’s geese [51], and other examples such as hand-reared Steller sea lions (*Eumetopias jubatus*) seeking human contact even after their release in the wild [52].

The relative influence of both factors (early deprivation from species-specific models and allospecific foster rearing) on species-specific social representation may depend upon the timing of this disrupted developmental trajectory (e.g., deprivation during specific periods for social learning results in long-term alterations of social behaviours [53]). Moreover, the importance of the timing may be particularly influenced by the species’ “agenda of development” (e.g., [54]). However, little is known on the amount of time and developmental milestones required with the parental model to build proper species-specific social representations, whether or not subsequent hand-rearing by humans takes place.

Cheetahs (*Acinonyx jubatus*), a vulnerable feline species (under the International Union for Conservation of Nature, IUCN Red List) [55], constitute a good model for investigating this question because of their relatively simple social system in which they have only one conspecific adult model during a “two-stage” development. Indeed, female cheetahs are solitary (except while with their dependent offspring) and raise their cubs alone [56,57,58]. The cheetah’s development is characterized by two different phases: (i) in the first stage (first two months of life), under predation pressure from larger predators, cubs remain hidden in a den, where they receive regular but temporary visits from their mother, who feeds them onsite; and, (ii) in the second stage, from around 2 months of age, cubs leave the den and follow their mother outside. They are then almost permanently with her, learning hunting skills amongst others, until they are around 18 months old, when the mother is generally separated from the litter [56,59]. Therefore, it is likely that the cheetah mother’s influence on their offspring varies according to the cubs’ developmental stage.

It is not uncommon that cheetah cubs become orphans before reaching independence, as adult cheetahs can be killed by larger predators or humans due to human–wildlife conflict [60,61,62]. Given their conservation status, orphaned young cheetahs, when found alive, are generally rescued in conservation centres where they are reared by humans. Cheetah juveniles may arrive in these centres at different stages of development, which means that they had experienced variable amounts of maternal “modelling” beforehand and also that they require different degrees of human care.

In the present study, we hypothesized that the timing of orphancy with regard to this two-step development is crucial for determining the cheetah’s perception of humans and of their characteristics. The limited number of interactions with a conspecific adult during the den-stage and subsequent intensive hand-rearing by humans may give rise to a higher degree of interest for humans, or even an integration of humans into their “social world”. On the other hand, cheetahs orphaned after den-age may have built a more complete species-specific representation that may override the impact of the moreover lighter foster care by humans. The long-term effects of early experiences have been revealed in earlier studies, which showed that adult captive cheetahs, hand-reared at a very early stage, discriminate the voices of familiar and unfamiliar humans [39], but also that the stage of development at which orphancy occurred affects vocal repertoire use and species-specific/interspecific auditory representation in wild-born orphaned captive cheetahs [14,63].

To test our hypothesis, we presented 14 wild-born captive-reared adult cheetahs with familiar and unfamiliar persons in a human motionless test. All cheetahs had been orphaned before the age of 6 months, either during the first or second phase of their development, and were living in the same conservation centre. We expected Early-Orphaned cheetahs to be more interested and more sensitive to human cues than Late-Orphaned cheetahs.

## 2. Materials and Methods

### 2.1. Study Site and Subjects

Human motionless tests were conducted at the conservation and research centre of the Cheetah Conservation Fund (CCF, Otjiwarongo, Namibia) in October–December 2023. CCF is an in-situ conservation centre that rescues wild-born cheetahs that have lost their mother when still dependent on maternal care. At the time of the study, CCF housed 24 adult and subadult resident cheetahs of both sexes (11 males and 13 females) aged between 1 and 14 years. They had been orphaned before six months of age. Adult individuals orphaned at the age of six or more months were not available for the study as they had been released back into the wild as per Walker et al. [64]. Of the 24 resident cheetahs, 10 were excluded on the basis of the following criteria: (i) they were housed too far away from the centre and thus did not have the same amount or regular experience with a large variety of human interaction (both familiar and unfamiliar), (ii) they were subadult individuals awaiting being released back into the wild and human contact was thus limited, and (iii) they were not used to being isolated in the small area where tests were conducted (see below).

The 14 individuals selected for the study were separated into two groups based on the moment of development in which they were rescued, as per Bouchet et al. [63]: (i) ‘Early-Orphaned’ cheetahs that were separated from their mother before 2 months of life (i.e., “den age”) (*N* = 4 males and 4 females); and (ii) ‘Late-Orphaned’ cheetahs that were separated from their mother between 2 and 6 months of life (i.e., post-den age but still dependent on mother) (*N* = 3 males and 3 females). All cheetahs were adults at the time of the study, and there was no difference in age between both groups (Early-Orphaned: 1–13 years; and Late-Orphaned: 2–14 years) (Supplementary Table S1).

Upon arrival at the CCF centre, Early-Orphaned cheetahs were kept in a specific nursery, where they were housed singly, with siblings, or with other cubs of similar age, and experienced different degrees of interaction with caretakers ranging from bottle to hand-feeding depending on the needs. Late-Orphaned cheetahs were also hand-fed, but required fewer human interventions and were kept in specific enclosures with siblings or same-age unrelated peers.

Adult cheetahs were housed in large outdoor enclosures (2–5 ha) with natural and artificial shelters and had access to a smaller enclosure used exclusively at feeding time (i.e., “feeding camp”, 64–206 m^2^). Cheetahs were fed with horse or donkey meat once a day, apart from one weekly day of fasting to mimic the natural habits of cheetahs. Clean water was provided ad libitum. All adult cheetahs were regularly involved in a mechanical lure course to provide them with physical exercise, where they were allowed to chase a piece of cloth that was attached to a 300 m line around a large enclosure.

Cheetahs were kept in groups of same-age/same-sex, related and/or unrelated individuals which, at the time of the experiments, had been stable for at least 1 year. No direct contact was possible between different groups, but groups of adjacent enclosures had visual and auditory contact. CCF centre was open every day between 9:00 a.m. to 5:00 p.m. to potential visitors. Cheetahs could experience a variety of encounters with humans: familiar caretakers who fed them daily and cared for them, including going into the enclosures when needed for medical reasons; interns and volunteers from different backgrounds who contributed to food preparation and distribution but did not go into the enclosures (no direct contact); and visitors who were present during controlled times of visits with an accompanying staff member, always at a distance, either at meal time or during the cheetah run, behind grids. Cheetahs were, therefore, used to the presence of a large variety of humans, both familiar and unfamiliar.

### 2.2. Experimental Procedure, Data Collection, and Analyses

Tests were conducted at 11:00 a.m. and 4:30 p.m. so as to have at least 1 h interval between testing and main activities with cheetahs involving the presence of humans around, such as feeding time in presence of visitors (12:00 on weekends and 14:00 p.m. on weekdays) or potential veterinary procedures. During the experiment, no humans were present around the cheetah except for the participating human and the experimenter (A.G.). Only one cheetah subject was tested at a time for a total testing time of 10 min per test. All tests were performed in the feeding camp, in which the focal individual could be separated from the rest of the group to avoid any potential influence on its reaction. The 14 individuals involved in the study were all used to being separated in this area for feeding and/or husbandry reasons. Each cheetah subject was involved in two trials, one with a familiar human and one with an unfamiliar human, at the same time of day but on different days (number of days separating two tests per subject = 16 ± 7.09). Seven subjects were tested, first, with the familiar human, and, second, with the unfamiliar one, and, for the seven others, it was the reversed order. Late- and Early-Orphaned subjects were evenly distributed between these two orders. The familiar humans involved were CCF staff members who had at least 1 year of experience with the cheetahs tested. Unfamiliar humans were individuals who had no experience with the cheetahs tested (i.e., new interns or CCF staff members from other departments). Both familiar and unfamiliar humans wore the CCF uniform, worn daily by all staff members, and were matched within the two categories by considering their sex and physical size.

Given the criteria for considering a human as being familiar, only seven familiar humans were involved (three men and four women), each participating once with a male and once with a female cheetah subject. The choice to test the same familiar human with a female and a male was related to earlier findings that female and male cheetahs may differ in their reactions to human presence (i.e., voices [14]). It was easier to recruit unfamiliar humans, and, therefore, 14 unfamiliar humans were involved, one per cheetah.

A test started when the human was in position close to the enclosure (at 1 m from the fence). The test was video-recorded using a camera (JVC Quad-Proof Everio R camera) held by the experimenter (A.G.) who was positioned 3 m behind the participating human. The human involved in the test was motionless, maintained a neutral behaviour, and did not initiate any visual or acoustic interaction with the cheetah for the entire duration of the test (i.e., arms alongside the body, no movement or gestures, looking in front of them and not at the cheetah). A summary of the procedure is given in Table 1.

Data were analysed by a single observer (A.G.) using Pot Player software (version 1.7). Based on earlier studies [42,65], the following behaviours were recorded: vocalizations (purrs and meows, as other call types are rare in captive context [63]), visual attention (differentiating between short glances [<1 s] and long gazes [>1 s] (as in [66]), excitation behaviours (i.e., self-grooming, yawning, scratching, and pacing) [65], and activity changes (e.g., lying down resting and then raising and starting to walk or raise the head and observe [67]) (see list and description of behaviours in Supplementary Table S2).

### 2.3. Statistics

Statistical analyses were performed on R software (R studio version 4.1.3, R Core Team 2021 [68]). Non-parametric statistics were conducted due to the non-normal distribution of the data and the small sample size. Statistics focused on potential differences depending on the developmental background of cheetahs (Early- or Late-Orphans) as well as influence of familiarity with human stimuli (Familiar or Unfamiliar). Wilcoxon tests were used to compare the behavioural differences of the subjects (separately for Late- and Early-Orphans) in the two conditions (Familiar vs. Unfamiliar). Mann–Whitney tests were conducted to compare individuals with different developmental backgrounds (Early- vs. Late-orphans), separately for Familiar and Unfamiliar test conditions. Results were considered as statistically significant if *p* ≤ 0.05.

## 3. Results

Cheetahs strongly differed in their responses to human presence and familiarity depending on their past developmental history.

**Figure 1:**
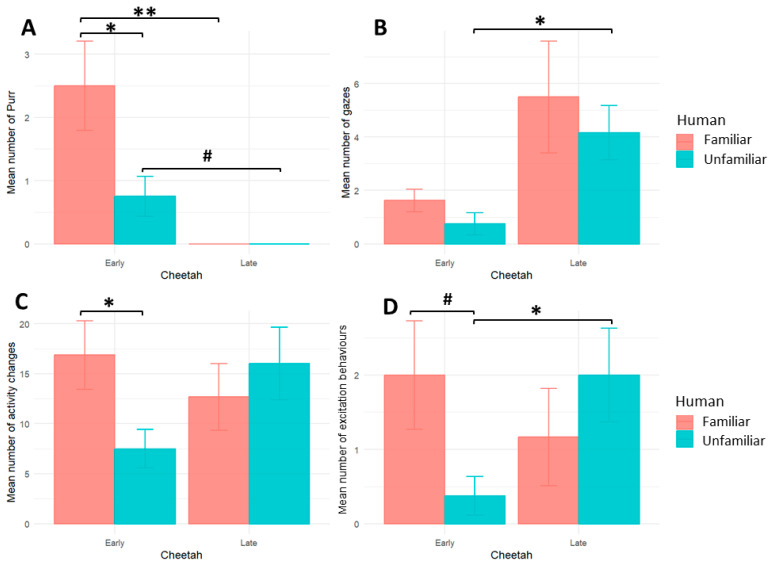
Bar plot (Mean ± S.D. per individual) showing the mean frequency of behaviours expressed in presence of Familiar (red) and Unfamiliar (blue) humans in both Early- and Late-Orphaned populations. (**A**) Purrs (Intra-population comparisons: Wilcoxon test; Inter-population comparisons: Mann–Whitney test, # *p* = 0.06, * *p* ≤ 0.05, ** *p* ≤ 0.01). (**B**) Gazes (Inter-population comparisons: Mann–Whitney test * *p* ≤ 0.05). (**C**) Activity changes (Intra-population comparisons: Wilcoxon test * *p* ≤ 0.05). (**D**) Excitation behaviours (Inter-populations comparisons Mann–Whitney test * *p* ≤ 0.05). In summary, intra-population differences were found only for Early-Orphaned cheetahs (more purrs and activity changes in presence of a familiar human than an unfamiliar human) and inter-population differences were less gazes and excitation behaviours in Early-Orphaned cheetahs than in Late-Orphaned cheetahs when in presence of an unfamiliar human.

Early-Orphaned cheetahs clearly discriminated between familiar and unfamiliar humans. Thus, they purred more (Figure 1A) and exhibited more activity changes (Figure 1C) when the human was familiar (Purrs: F: 2.5 ± 2, NF: 0.75 ± 0.88; Wilcoxon test, V = 26, *p* = 0.05, effect size = 0.72, Activity changes: F: 16.87 ± 9.70, NF: 7.5 ± 5.39, V = 34.5, *p* = 0.02, effect size = 0.81). Late-Orphaned cheetahs, instead, did not purr regardless of the human category (Wilcoxon test not applicable, as all data were tied and zero; Figure 1A).Late-Orphaned cheetahs also failed to show any statistically significant difference in activity changes according to human’s familiarity (Figure 1C) (Activity changes: F: 12.66 ± 8.18, NF: 16 ± 8.89, V = 6, *p* = 0.78).

Furthermore, there were no statistically significant differences for either Early- and Late-Orphaned cheetahs according to human familiarity in visual attention including both glances and gazes (Figure 1, Supplementary Table S2).

When comparing the responses between cheetah categories in their reactions to humans, clear differences appeared between Early- and Late-Orphaned cheetahs. None of the Late-Orphaned cheetahs produced purrs for any of the humans, while the Early-Orphaned did, especially for the familiar humans (Mann–Whitney test, Familiar: Early vs. Late W = 45, *p* = 0.004, effect size = 0.77; Unfamiliar: Early vs. Late W = 36, *p* = 0.06, effect size = 0.52) (Figure 1A). Moreover, Early-Orphaned cheetahs gazed less often at the human (Figure 1B) and expressed less excitation behaviours than Late-Orphaned cheetahs when in the presence of an unfamiliar human (Gazes: Early vs. Late W = 6, *p* = 0.01, effect size = 0.64; Excitation behaviours: W = 8, *p* = 0.03, effect size = 0.59) (Figure 1D), while no statistically significant difference was found for familiar humans, possibly because of the high individual variations observed, especially in the Late-Orphaned cheetahs (Gazes: W = 13.5, *p* = 0.498) (Figure 1B,D). Other behavioural measures showed no statistically significant differences according to the cheetah or human category, such as meows, glances, or activity changes (Supplementary Table S3).

## 4. Discussion

The results of this study strongly suggest that cheetahs’ reactions towards a human presence are influenced by their developmental background. Only Early-Orphaned cheetahs responded differentially to familiar and unfamiliar humans, showing a clear discrimination. They also performed purrs, considered as a social affiliative behaviour, towards both categories of humans, suggesting some generalization of the positive perception from familiar to unfamiliar humans. Conversely, Late-Orphaned cheetahs did not produce any purrs and showed no behavioural difference according to the human familiarity.

Purring in cheetahs is generally considered as a social affiliative signal, expressed during positive encounters (e.g., mutual grooming) or relaxed moments (e.g., after a meal) [56,57], whereas, in captive individuals, purrs are also emitted towards their human keepers [69,70]. This interspecific use of purrs may be a consequence of captive cheetahs’ developmental backgrounds, in which human hand-rearing is common practice [71,72,73]. Purrs are often emitted in association with positive social interactions and bonding at juvenile stages in felid species, such as in the domestic cat that purrs as a form of contentment or request for care from the mother [74] or emits purrs redirected to humans to solicit care [75,76,77]. Hence, the purring of Early-Orphaned cheetahs toward humans may reflect social affiliation with humans as a consequence of the parental bond being replaced/supplemented by their human caretakers and/or a form of neoteny resulting from early deprivation from their conspecific primary adult model [6]. Interestingly, cheetahs that had longer experiences with their mother (Late-Orphaned), although showing attention towards both categories of humans in the form of gazes, activity changes, and excitability behaviours, never emitted purrs in the presence of humans, thus suggesting that a prolonged experience with conspecifics can override the effects of the subsequent human hand-rearing on cheetahs’ social representations. In fact, it has been shown that Late-Orphaned cheetahs have a very low use of purrs compared to early orphans [63], including in the presence of humans [78].

Moreover, our results also suggest a potential difference between both developmental groups in terms of human recognition. Even if Early-Orphaned cheetahs purred with humans overall, they appeared sensitive to humans’ familiarity as they preferentially purred in the presence of familiar humans. This is in line with previous findings on captive hand-reared cheetahs that recognize familiar humans by just hearing their voices [39] and presents a similarity with domestic cats who also emit purrs when their human companion approaches [79]. The ability to discriminate between humans is associated with the enhanced attention of the animals towards humans with whom interactions regularly occur. In fact, it has been shown that captive animals are attentive to their familiar human caregivers [80,81,82,83,84] and actively monitor their actions, possibly to catch cues predicting the next potential interaction [85,86,87,88,89,90]. Here, Early-Orphaned cheetahs had extensive interactions with humans at very early stages of their development, and, so, humans probably acquired a certain positive valence as “primary caregivers” which may have enhanced the discrimination of familiarity. Beyond purring more in the presence of familiar humans, Early-Orphaned cheetahs also presented more activity changes in the presence of the familiar human, possibly in anticipation of interactions, as was shown in early-hand-reared cheetahs in the study of Leroux et al. [39].

The lack of statistically significant differences in the reactions seen in Late-Orphaned cheetahs according to humans’ familiarity may be explained by the following: (1) Late-Orphaned cheetahs may be unable to discriminate between different humans, as they did not build a multisensory representation of familiar caretakers at an early age, or, (2) the familiarity of humans is not relevant for them as all humans are in a same “heterospecific” category, being exposed to large numbers of staff and volunteers acting as caretakers (e.g., providing feeding), thus with no difference in associated valence. The overall lack of differential responses between both categories of humans does not appear to result from a lack of interest in humans in general, as their levels of attention and activity changes were high in the presence of both familiar and unfamiliar humans, indicating that Late-Orphaned cheetahs are indeed sensitive to the human presence and “alert” about it. But there were also high individual variations and it may be that precise experiences at a very early stage are determinant for building social representation and the lack thereof precludes individual recognition for, in the present case, the foster heterospecific caretakers. This may also extend to individuals from the same species, as it has been shown in felids that early deprivation from adult conspecifics results in altered social representation and skills [8,91].

Taken together, these results suggest an interplay between the timing of maternal loss and the amount of care provided by a foster species, i.e., humans, in this case. Moreover, our findings indicate that the timing in which an emotional bond is created with the primary caregiver (humans in the case of Early-Orphaned cheetahs and the mother in the case of Late-Orphaned cheetahs) is crucial in determining the reaction towards the species of the early caregiver throughout life. This is in line with the observations of greylag geese (*Anser anser*) hand-reared from the egg stage, which considered humans with the same valence as conspecific parents [92] and Steller sea lions hand-reared within the first 24–48 h, that developed an attachment towards humans that led to preferentially seeking contact with humans over conspecifics even after the release back into the wild [52]. Early-hand-reared chimpanzees, even after their successful re-introduction in a social group and social integration with conspecifics, still showed social behaviours directed towards humans [6]. Hence, the amount of time spent with the parental model before human hand-rearing appears crucial in building proper social representations and behaviours (e.g., [53]), but this “agenda” of social development also depends on the developmental trajectory of each species [54]. In the present case, the two-step development of cheetahs may be determinant in building this agenda. Further studies on other species presenting a “den stage” would help enlighten the debate on the long-term effects of the timing of early experiences on species-specific representations.

## 5. Conclusions

The finding that Early-Orphaned cheetahs differed strikingly in their reactions to the presence of familiar and unfamiliar humans, with Early-Orphaned showing clearly more sensitivity and affiliation with familiar humans, provides new insights on human and wild animal relationships. Most of all, it emphasizes the importance of their timing in possibly shaping or altering potential species-specific influences, with consequences for animals’ conservation and management. It also provides a new scenario for how early-life social experience and its timing might influence the development of social and interspecies representation in non-domesticated species.

## Figures and Tables

**Table 1:** Step-by-step description of the experimental procedure of testing.

Step	Time	Description
1. Selection of Familiar (F) vs. Unfamiliar (NF) Human	Preparatory phase	Familiar humans are CCF staff with ≥1 year of direct experience with the study cheetahs. Unfamiliar humans are new interns or CCF staff that had no experience with the study cheetahs.
2. Cheetah Separation	At least 30 min before the test started	Each cheetah is separated from the group in the feeding camp to avoid external influences.
3. Start of experiment	Conducted at 11:00 a.m. or 4:30 p.m.	The trials are conducted at two specific times during the day (11:00 a.m. and 4:30 p.m.), ensuring that the cheetahs are not influenced by other human activities (e.g., feeding).
4. Human Positioning	Start of the test	The human participant stands 1 m from the fence, remaining motionless and neutral.
5. Testing Period	10 min per test	Each test lasts 10 min.
6. Repetition of test (F to NF or NF to F)	Spacing between tests: 16 ± 7 days	Seven subjects were tested first with the familiar human, and the remaining seven were tested first with the unfamiliar human (evenly distributed between Early- and Late-Orphaned cheetahs).

## Data Availability

The raw data underlying the findings of this article will be made available by the authors upon request.

## Supplementary Materials

The following supporting information can be downloaded at: https://www.mdpi.com/article/10.3390/ani14223223/s1, Table S1: List of cheetahs involved in the study; Table S2: List of behaviours performed by cheetahs during the human motionless tests; Table S3: Results of the statistical tests according to Human familiarity.
