# Supplementary material for: Agenda of Early Life Experience and Its Association with Sensitivity to Human Presence and Familiarity in Wild-Born Orphaned Captive Cheetahs
Source: Animals (Basel). 2024 Nov 10;14(22):3223. doi: 10.3390/ani14223223 (PMC11591097; doi:10.3390/ani14223223)
Supplement: Supplementary file 1 [file animals-14-03223-s001.zip › animals-3285680-supplementary.pdf]

Supplementary Table S1

List cheetahs involved in the study divided depending on whether they were Early- or Late-Orphaned individuals.

EARLY – ORPHANED CHEETAHS			
**ID**	**AJU**	**SEX**	**AGE**
Dominic	AJU1776	M	5 years
Khaleesi	AJU2052	F	2 years
Kora	AJU2107	F	20 months
Peter	AJU1601	M	14 years
Rocket	AJU1923	M	4 years
Senay	AJU1600	F	14 years
Teja	AJU2105	M	20 months
Tigerlily	AJU1603	F	14 years
**LATE-ORPHANED CHEETAHS**			
**ID**	**AJU**	**SEX**	**AGE**
Aurora	AJU1641	F	12 years
Jev	AJU2100	M	2 years
Katiti	AJU1772	M	6 years
Koya	AJU1749	M	6 years
Marissa	AJU2059	F	13 years
Rainbow	AJU1640	F	12 years

Supplementary Table S2

List of behaviours performed by cheetahs during the human motionless tests (adapted from Leroux et al. (2018) and Bouchet et al. (2022))

**Behaviour**	**Description**
**VOCAL BEHAVIOURS**	
Purrs	Low-pitched vocalization with pulsed structure, produced continuously during both inhalation and exhalation phases, often associated with affiliative or relaxed behavioral contexts.
Meows	Higher-pitched tonal vocalization, used mainly for calling over or contact-care solicitation.
Other call types	Rare vocalizations, see Bouchet et al. (2022) for specific definitions.
**VISUAL ATTENTION**	
Glance	Brief visual attention (< 1 sec) towards the stimulus.
Gaze	Sustained visual attention (> 1 sec) towards the stimulus.
**EXCITATION BEHAVIOURS**	
Self-grooming	Subject grooms its own body (e.g. licking, cleaning).
Yawning	Subject opens its mouth widely while inhaling then closes it while exhaling deeply.
Scratching	Subject uses its limbs or mouth to scratch different parts of its body.
Pacing	Repetitive walking in a fixed pattern, that is back and forth on the same route.

Behaviour	Description
**ACTIVITY CHANGES**	Any activity change performed such as from lying down to walking, observing to gazing, resting to observing…

Supplementary Table S3

Table showing the results of the statistical tests according to human familiarity.

**Behaviour**	**Statistical Test (V, p-value)**
**Early-Orphaned Familiar versus Unfamiliar**	
**Purr**	V = 26, **p = 0.05**
**Activity changes**	V = 34.5, **p = 0.02**
Gazes	V = 16.5, p = 0.235
Excitation behaviours	V = 10, p = 0.09
Glances	V = 6, p = 0.17
Meows	V = 1.5, p = 1
**Late-Orphaned Familiar versus Unfamiliar**	
Purr	all values were 0 for both conditions (Purr: F: 0 ± 0, NF: 0 ± 0). Wilcoxon test non applicable
Gazes	V = 10, p = 0.586
Excitation behaviours	V = 2, p = 0.34

Glances	V = 16.5, p = 0.24
Meows	V = 0, p = 0.37
**Early-Orphaned versus Late-Orphaned Familiar**	
**Purr**	**W = 45, p = 0.004**
Gazes	W = 13.5, p = 0.18
Glances	W = 18.5, p = 0.51
Excitation behaviours	V = 29.5, p = 0.49
Meows	W = 27, p = 0.47
Activity changes	W = 29, p = 0.56
**Early-Orphaned versus Late-Orphaned Unfamiliar**	
**Gazes**	**W = 6, p = 0.01**
**Excitation behaviours**	**W = 8, p = 0.03**
Glances	W = 18, p = 0.44
Meows	W = 18, p = 0.32
Activity changes	W = 10, p = 0.08
Purr	W = 36, p = 0.06
